# Anthropogenic Disturbances Eroding the Genetic Diversity of a Threatened Palm Tree: A Multiscale Approach

**DOI:** 10.3389/fgene.2019.01090

**Published:** 2019-11-07

**Authors:** Leiza Aparecida Souza Serafim Soares, Eliana Cazetta, Larissa Rocha Santos, Daniele de Souza França, Fernanda Amato Gaiotto

**Affiliations:** ^1^Applied Ecology and Conservation Lab, Programa de Pós-Graduação em Ecologia e Conservação da Biodiversidade, Universidade Estadual de Santa Cruz, Ilhéus, Brazil; ^2^Laboratório de Marcadores Moleculares, Centro de Biotecnologia e Genética, Universidade Estadual de Santa Cruz, Ilhéus, Brazil

**Keywords:** landscape genetics, tropical rainforest, conservation, threatened species, molecular marker

## Abstract

Habitat loss and the illegal exploitation of natural resources are among the main drivers of species extinction around the world. These disturbances act at different scales, once changes in the landscape composition and configuration operate at large scales and exploitation of natural resources at local scales. Evidence suggests that both scales are capable of triggering genetic erosion in the remaining populations. However, most of the studies so far did not evaluate simultaneously the effects of these disturbances on genetic diversity and structure of plants. In this study, we used a multiple scale approach to empirically evaluate the impacts caused by local and landscape scale disturbances in the genetic diversity and structure of an endangered palm tree, *Euterpe edulis*. We sampled and genotyped with microsatellite markers 544 juveniles of *E. edulis* in 17 fragments of Atlantic Forest in Brazil. In addition, we estimated the local logging rate and the forest cover and isolation at landscape scale. We found that the palm populations have not undergone any recent bottleneck events and that only logging intensification had affected the fixation index and the number of private alleles. Additionally, we did not detect any evidence of spatial genetic structure or genetic divergence associated with environmental disturbance variables at different scales. However, we identified distinct genetic clusters, which may indicate a reduction of gene flow between fragments that were previously a continuous habitat. Our results show that local disturbances, which act directly on population size reduction, such as logging, modified the genetic diversity more rapidly, whereas genetic structure is probably more influenced by large-scale modifications. In this way, to maximize the conservation efforts of economically exploited species, we recommend to increase the inspection to reduce the illegal exploitation, and reforestation of degraded areas, in order to increase the gene flow in Atlantic Forest fragments.

## Introduction

The conversion of natural environments into anthropogenic landscapes is one of the main causes of biodiversity loss worldwide (Laurance et al., 2014; Newbold et al., 2015; Miraldo et al., 2016). Mounting evidence shows the pervasive effects of habitat loss on several taxonomic groups (Mortelliti et al., 2010; Ferreira et al., 2015; Muylaert et al., 2016; Rocha-Santos et al., 2017). The remaining forest patches are being reduced and isolated with negative consequences for species diversity. In this context, the remaining populations are prone to gene flow decrease, alleles loss due to genetic drift, and inbreeding depression. The consequence of these genetic changes might be a lower ability to adapt to further environmental changes (Bijlsma and Loeschcke, 2012; Fountain et al., 2016; Rhoads et al., 2017; Browne and Karubian, 2018). Several studies have corroborated this prediction (Dixo et al., 2009; Zhang et al., 2012; Wood et al., 2017), however, the intensity and the velocity of the response to impacts vary according to life history and environmental characteristics (Vranckx et al., 2012; Lino et al., 2019).

In human-modified landscapes, species of economic value are also locally vulnerable due to (i) the direct removal of individuals or (ii) the indirect exploitation of resources, such as seeds, impairing the regeneration capacity of these species (Homma, 1992; Peres et al., 2003). Acute disturbances, such as logging, are capable of causing severe demographic depletion over generations (Richardson and Peres, 2016) and increase the risk of genetic erosion in the remaining populations (Shaanker et al., 2003; Shaanker et al., 2004; Dai et al., 2018). Theoretical and empirical studies have observed reductions in allele number and heterozygosity and an increase in spatial genetic distance in exploited populations (Cruse-Sanders et al., 2005;Lacerda et al., 2008; Sebbenn et al., 2008). However, investigations that simultaneously assessed the individual impact caused by landscape and local scale disturbances are scarce (but see González-Fernández et al., 2019).

The combination of anthropogenic pressures at the landscape and local scale is striking in the tropics. The tropics stand out worldwide due to the high rates of species biodiversity and endemism, but the increasing human population size and forest loss is a growing threat (Hansen et al., 2013; Lewis et al., 2015). In this scenario, understanding the individual impact of different types of disturbance on the genetic diversity of exploited species could better direct conservation efforts. For this reason, in this study, we used a multi-level approach to investigate the effects of anthropogenic disturbances at the landscape and local scale on the genetic diversity and structure of *Euterpe edulis* populations. This species is native to the Atlantic Rainforest (Reis et al., 2000a), a biome that currently conserves only 12% of its original coverage (Ribeiro et al., 2009). Moreover, this palm is in the list of the Brazilian species threatened with extinction because of the population decline recorded in the last decades (Martinelli and Moraes, 2013). The species has great economic importance due to the illegal harvest to the commercialization of the apical meristem (Galetti and Fernandez, 1998; Matos and Bovi, 2002). The harvest culminates in the death of individuals, as there is no regrowth after cutting (Ferri and Cavalcante, 1997). In the tropics, changes in the landscape composition and configuration, such as forest cover loss and isolation provide more accessibility to forest resources, increasing illegal hunt and logging (Tabarelli et al., 2004). These activities might in turn, negatively affect seed dispersal by vertebrates and seedling recruitment (Peres and Palacios, 2007; Gutiérrez-Granados, 2011). Using a multiscale inference approach, we evaluated how landscape composition and configuration, measured at several spatial scales, and local disturbances, affect the genetic diversity of *E. edulis*. We predicted that estimates of genetic diversity and structure would be strongly influenced by the synergetic effects of disturbances acting at different scales. Thus, models containing a combination of landscape and local variables would better explain the genetic diversity of the species.

## Materials and Methods

### Study Area and Sites Selection

The study region was located in the Atlantic Rainforest in Southern Bahia State, Brazil. Deforestation in the region started in the mid-1980’s and was intensified in the 1990s during the cocoa crisis, the main economic product at that time (Rocha, 2006). The region presents some of the last remnants of the northeastern Atlantic Forest (Araújo et al., 1998) and still harbors a large number of flora and fauna species, including several endemics (Thomas et al., 1998). The land use history resulted in a mosaic of forest patches in different successional stages immersed in a matrix of pasture, rubber and eucalyptus plantations, and cacao agroforests (Landau, 2003; Sambuichi, 2003; Landau et al., 2008). The native vegetation is tropical lowland rainforest and the climate is classified as Af, according to Köppen, hot and moist, without a distinct dry season (Gouvêa, 1969).

**Figure 1:**
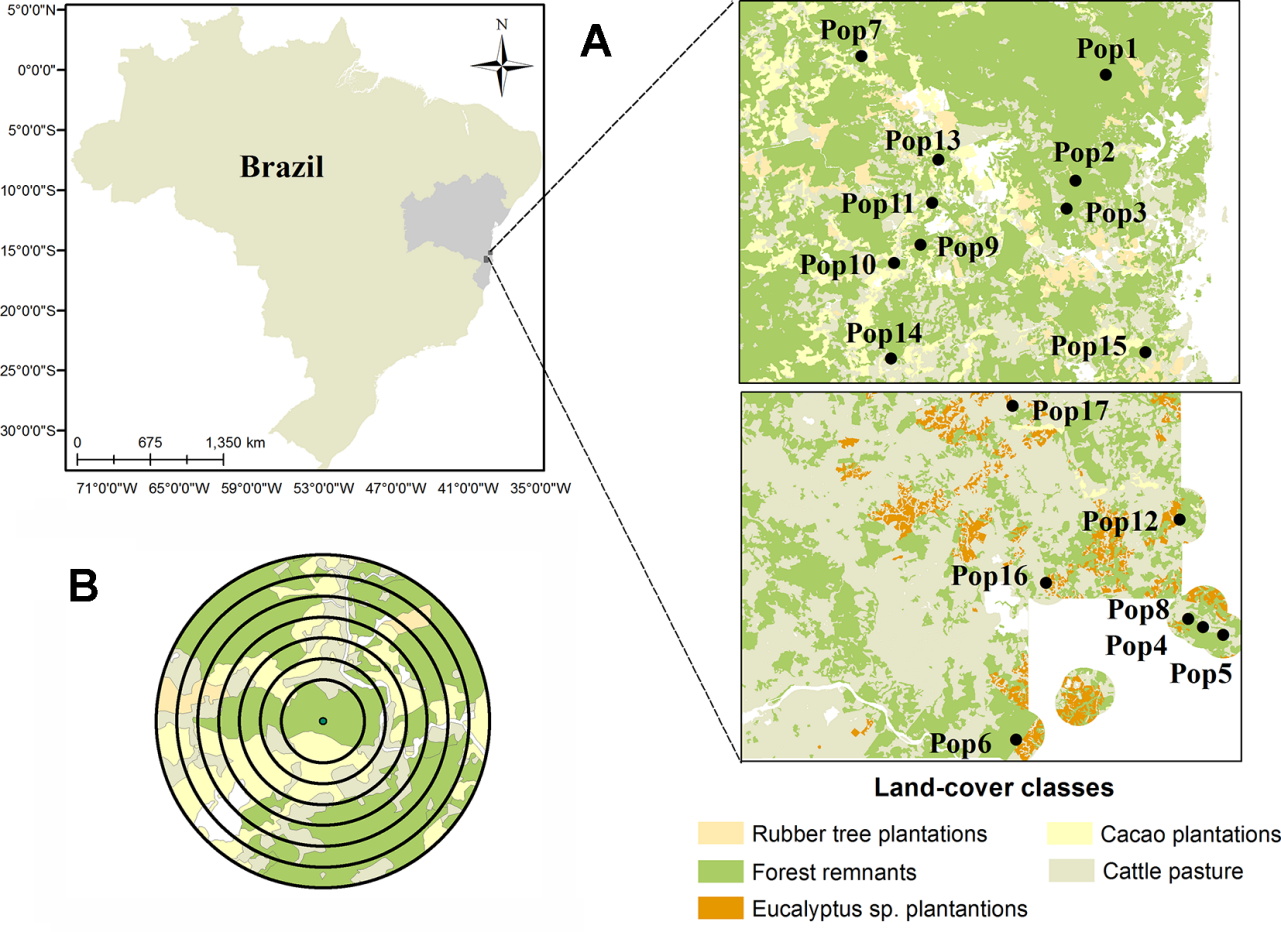
Location of the 17 forest fragments in which the populations of *Euterpe edulis* were sampled in the Atlantic Forest of southern Bahia. **(A)** Distribution of fragment center points (circle) and characterization of the main forms of land use in the region; **(B)** Example of the seven buffers created around the fragment’s center point for landscape metric calculations.

After an intense process of ground-truthing, we mapped the land-use of 3,470 km^2^ using high-resolution satellite images (i.e. RapidEye from 2009-2010, QuickBird and WorldView both from 2011; with resolutions of 5, 0.5, and 0.6 m, respectively). We avoided to sample in Montane and Restinga Forests, which resulted in a subdivision into two regions (north and south, **Figure 1A**) mainly due to a sandy stripe between them. The history of deforestation and land use are different between the two regions. The matrix of the north region is more heterogeneous and in the south region the matrix is mainly dominated by pastures. However, the two regions show similar soil, topography and vegetation types (Thomas et al., 1998). The main land-use classes were classified as: forests (mature and secondary native forest), cattle pasture, and plantations of cacao, rubber tree, and *Eucalyptus* sp.) From the map, we selected 58 possible forest fragments adopting as exclusion criteria highly demanding access areas, and indigenous lands. Then, we used previous studies in the region to select a subset of 17 forest fragments, at least 2 km apart, which spanned a large range of landscape-scale forest cover (**Table S1**), and for which the occurrence of *E. edulis* was known (Santos et al., 2015; Soares et al., 2015; Benchimol et al., 2017).

Our sampling occurred between 2014 and 2016, and we used Google Earth to evaluate substantial changes in land use cover during the period between mapping (2009–2011) and data collection. After finding stability in land use cover during this period, we performed all analyzes with forest cover calculations obtained by the high-resolution mapping.

### Focal Species and Sampling

*Euterpe edulis* is a monoecious palm tree, with annual and predominantly crossed reproduction (Mantovani and Morellato, 2000; Gaiotto et al., 2003; Castro et al., 2007). The flowers are abundant in nectar and pollen and attract a wide variety of insects (Reis et al., 2000b). Despite this, its pollination is mainly performed by bees belonging to different taxonomic groups such as Meliponini, Euglossini, and Honeybees (Reis et al., 2000b; Dorneles et al., 2013; Santos et al., 2018a; Santos et al., 2018b). *E. edulis* fruits have a pulp rich in lipid and fiber (Silva et al., 2013; Da Silva et al., 2014) and are used as food by 58 bird species and 20 mammal species (Galetti et al., 2013). However, the main seed dispersers are large frugivores such as toucans and cotingas. Small birds such as thrushes are especially important in defaunated areas (Reis and Kageyama, 2000; Galetti et al., 2013; Santos et al., 2018a; Santos et al., 2018b).

We randomly established three 50 × 10 forest-plots in each of the 17 forest fragments. Plots were located at least 50 m apart from each other and from the nearest edge. In each forest plot, we sampled all *E. edulis* juvenile (i.e. individuals with pinnate leaves, height ≤0.15 and >1.00 m) based on the categories proposed by Silva et al. (2009). Subsequently, all individuals were georeferenced and had a leaf tissue sampled. We chose the juvenile ontogenetic stage because the genetic parameters of the early developmental stages better represent the effects of recent environmental disturbances (Vranckx et al., 2012). In addition, juveniles might show more consistent responses to anthropogenic disturbances because they exhibit higher numerical stability in terms of population fluctuation than seedlings (Conte et al., 2000). All collected individuals were numbered and later, 32 individuals were drawn in each of the 17 population for genotyping. This procedure resulted in a total sampling of 544 individuals. The determination of the number of individuals per population was based on a previous study that detected little benefit on the accuracy of allele frequencies and diversity estimates for microsatellite loci above 30 individuals (Hale et al., 2012).

### DNA Extraction and Genotyping

DNA extraction followed the CTAB protocol (Doyle and Doyle, 1987). We genotyped all *E. edulis* individuals using 17 nuclear microsatellite markers developed for the species (Gaiotto et al., 2001). Multiplex PCR reactions were performed in a Veriti™ Thermal Cycler (Applied Biosystems, Foster City, CA) with two triplex combinations (EE43, EE45, EE52 and EE47, EE59, EE63) and two duplex combinations (EE2, EE32 and EE8, EE23). We also performed single locus PCRs to the following markers: EE3; EE5; EE9; EE15; EE25; EE48; and EE54. Subsequently, we submitted only the EE5 marker product to the individual electrophoresis system, while the PCR products of the other markers were submitted to the multiload electrophoresis system adapted from Gaiotto et al. (2003) and were organized with the following combinations: (i) Pentaload I, (EE2, EE32, EE8, EE23, EE3); (ii) Pentaload II (EE43, EE45, EE52, EE48, EE54); (iii) Tetraload (EE47, EE59, EE63, EE25); and (iv) Biload (EE9 and EE15). The mix submitted to the electrophoresis was composed of 2 µl of the PCR product (or of the mix of the PCR products for the cases of the multiload system), 0.2 µl of GeneScan^™^ 500 Liz^™^ (Applied Biosystems, Thermo-Fisher Scientific, Inc., Waltham, MA, USA) and 7.8 µl of deionized formamide (Applied Biosystems). Later, we performed genotyping on the ABI 3500 Genetic Analyzer (Applied Biosystems, Foster City, CA). The sizing of fragments was obtained with GeneMarker^®^ software 2.2 (SoftGenetics, State College, PA, USA).

### Population Genetics

We evaluated the Hardy–Weinberg equilibrium within each sample population with the package DiveRsity (Keenan et al., 2013) on the R environment 3.5.2 (R Core Team, 2018) and tested the linkage disequilibrium between all pairs of loci through FSTAT 2.9.3 (Goudet, 2001). The null alleles were corrected using the method of Oosterhout (van Oosterhout et al., 2006), in MICRO-CHECKER software v. 2.2.3 (van Oosterhout et al., 2004) after the results of 999 bootstrap simulations with a confidence interval of 95%. After null alleles correction, we perform the estimations of all genetic parameters. The DiveRsity package was used to calculate observed and expected heterozygosity (H_O_ and H_E_, respectively), allelic richness, and fixation index with a 95% confidence interval estimated by 999 bootstrap simulations. In addition, we used the GENALEX 6.5 program (Peakall and Smouse, 2012) to calculate the number of private alleles in each population. Considering that detection of private alleles may be biased by sample insufficiency, we acknowledge that this estimate might include alleles that occur exclusively in a single population but also those presented in low frequencies (<0.05) that could not be detected. Finally, we used the software BOTTLENECK v.1.2.02 (Cornuet and Luikart, 1996; Piry et al., 1999) to investigate the occurrence of recent genetic bottlenecks in the populations sampled. We chose the two-phase mutation model, due to it is the most recommended for microsatellite data (Piry et al., 1999; Williamson-Natesan, 2005). We fitted this model with 95% of single-step mutations and 5% of mutations of multiple steps, as suggested by Piry et al. (1999). Subsequently we used the Wilcoxon test with 5,000 iterations to evaluate the occurrence of excess heterozygosity.

### Landscape Metrics and Local Variable

To evaluate whether the genetic diversity of *E. edulis* is influenced by anthropogenic disturbances, we related the genetic estimates with landscape and local attributes. At the landscape scale, we evaluated metrics of landscape composition and configuration that might affect genetic diversity and structure (Balkenhol et al., 2013; Jackson and Fahrig, 2016). These landscape attributes were calculated using the program FRAGSTATS v4.2.1.603 (McGarigal et al., 2002) for seven buffers of different radii sizes, ranging from 0.5 to 2 km, each 250 m, from the central plot in each forest fragment (**Figure 1B**). The highest radius was chosen because it covers the foraging distances of important large seed dispersers of *E. edulis*, such as toucans, which have a medium-sized seed dispersal distance ranging from 269 to 449 m (Holbrook, 2011). In addition it also includes different foraging distances reported for potential pollinators of *E. edulis*, such as *Plebeia doryana* (maximum 540 m) and *Apis mellifera* that can reach distances of hundreds of meters of foraging (Zurbuchen et al., 2010). We evaluated (i) forest cover (a proxy of habitat amount) as landscape composition descriptor and (ii) mean patch size, proximity index, and edge density as landscape configuration descriptors. For all scales, forest cover was calculated as the area occupied by the sum of mature and secondary native forests divided by the total landscape area. We used mature and secondary forests as a proxy of habitat because natural populations of *E. edulis* occurred in both types of forests. The calculation of all landscape configuration metrics was performed at the class level and was based only on the areas occupied by forest remnants within each landscape. Then, we evaluated the Pearson correlation among all landscape variables. We excluded from the subsequent analyses mean patch size and edge density due to the high correlation with forest cover amount in more than one spatial scale (r > 0.4) (**Table S2**).

At the local scale, we estimated logging activity as a disturbance variable related to *E. edulis* harvest. We used logging as a proxy of harvesting because both activities are intensified in anthropogenic landscapes and close to urban centers where they become one of the main income sources for local populations (Shaanker et al., 2003; Tabarelli et al., 2004). We also opt to estimate logging because the record of the illegal palm harvest is very scarce due to the rapid stipe decomposition when compared with hardwood trees (personal observation). The logging estimative in each fragment was made in 2014 when we evaluated a 100 × 8 m plot, distant at least 50 m from the closest edge. In all plots, we counted the wood stumps with the diameter at ground level ≥20 cm.

### Data Analysis

#### Genetic Diversity

To evaluate the spatial scale to which the environmental context influenced the genetic diversity (scale of effect) we used the multifit function (Huais, 2018) and generalized additive models (GAM: Hastie and Tibshirani, 1990). We related simultaneously the genetic attributes to the forest cover and proximity index quantified on seven spatial scales (buffers with 0.5, 0.75, 1.0, 1.5, 1.25, 1.75, 2.0 km radius). In addition, we evaluated the spatial autocorrelation of the residual models using the selected scale by applying a Moran I index (Fortin and Dale, 2005) (**Table S3**). We did not find any spatial autocorrelation in our data (**Supplementary Material**), hence it was not necessary to consider the spatial structure in further analyses.

We performed GAM to capture linear and nonlinear relationships between variables. All possible additive combination of independent variables (landscape and local attributes) and the genetic parameters were tested, totaling seven models. We tested for the concurvity among predictors i.e. the nonparametric counterpart of multicolinearity in linear regressions. We also included a null model containing only the intercept. Finally, we performed a model selection approach using the Akaike Information Criterion corrected for small sample sizes (AICc; Burnham and Anderson, 2002) for each one of the genetic parameters. Models with ΔAIC values ≤ 2.00 were considered to be equally plausible. But we chose the most parsimonious model, using the criterion of at least weight among plausible models.

#### Population Structure

The genetic differentiation among populations of *E. edulis* was investigated through three distinct approaches: we used the DiveRsity package to calculate the Gst-statistics (Hedrick, 2005) and Wright’s *F*_ST_ The statistical significance of these two analysis was evaluated by 1,000 randomizations with a confidence interval of 95%. The genetic differentiation measures estimated by Gst values are more suitable for microsatellite locus than those generated by Fst because they standardize the bias generated by the polymorphic character of these markers (Hedrick, 2005). However, Meirmans and Hedrick (2011)recommend that Fst values be presented in current studies to enable comparisons with older studies. The third approach of the genetic differentiation used in our study was the discriminant analysis of principal components (DAPC: Jombart et al., 2010) performed by the Adegenet package in R (Jombart, 2008). This analysis creates a model in which the genetic variation is partitioned between groups and within groups with the objective of maximizing variation between groups (Jombart and Collins, 2015). We initially used the K-means algorithm of the “find cluster” function and retained all the major components to detect evidence of genetic clusters in our populations (**Figure S1A**). We then applied the Bayesian Information criterion (BIC) to infer the best number of clusters by identifying the lowest BIC value (**Figure S1B**). Subsequently, we used the cross-validation function (Xval.Dapc) to identify the ideal number of principal component (PCs) to be retained based on the least mean square error after 1,000 stratified random simulations (**Figure S1C**). After obtaining this result, we performed the DAPC, which in the first moment transformed the genetic data using principal component analysis and then submitted the number of retained PCs to linear DAPC.

## Results

### Genetic Diversity Within Populations

Our results did not show evidence of genotypic linkage disequilibrium in any of the pairs of loci. In addition, we detected the presence of null alleles in all loci with frequency varying from 0.5 to 16%. The average of genetic parameters estimated with original or corrected data for null alleles lead to similar results (data not shown). However, we opted to show all results based on the corrected data for null alleles (**Table 1**). We detected a total number of 347 alleles in the 17 loci by the 544 juvenile individuals from all 17 *E. edulis* populations evaluated. We used highly polymorphic microsatellites and the number of alleles generated for each marker ranged from 7 (EE43) to 33 (EE52), with a mean of 20.4 alleles for loci.

**Table 1:** Genetic estimates and genetic bottleneck signature of 17 *E. edulis* populations.

Population	Ap	Ar	H_O_	H_E_	*f* (CI 95%)	TPM	Mode-shift
Pop1	1	8.19	0.66	0.68	0.022 (−0.034; 0.072)	1	L-shaped
Pop2	6	8.4	0.67	0.71	0.05 (−0.01; 0.105)	0.983	L-shaped
Pop3	7	8.38	0.69	0.72	0.036 (−0.015; 0.085)	0.997	L-shaped
Pop4	1	8.11	0.68	0.7	0.027 (−0.02; 0.069)	0.998	L-shaped
Pop5	3	7.58	0.6	0.65	0.08 (0.016; 0.135)	0.999	L-shaped
Pop6	11	7.35	0.63	0.65	0.041 (−0.025; 0.100)	0.996	L-shaped
Pop7	9	8.24	0.67	0.71	0.049 (−0.009; 0.096)	0.964	L-shaped
Pop8	4	8.66	0.74	0.76	0.027 (−0.018; 0.072)	0.993	L-shaped
Pop9	10	8.79	0.69	0.75	0.079 (0.025; 0.127)	0.998	L-shaped
Pop10	0	8.55	0.7	0.73	0.043 (−0.011; 0.096)	0.993	L-shaped
Pop11	3	8.72	0.7	0.75	0.072 (0.02; 0.122)	0.96	L-shaped
Pop12	5	8.44	0.72	0.76	0.057 (0.011; 0.100)	0.991	L-shaped
Pop13	3	9.11	0.74	0.76	0.036 (−0.03; 0.101)	0.997	L-shaped
Pop14	2	7.71	0.6	0.65	0.066 (0.014; 0.116)	1	L-shaped
Pop15	4	6.41	0.71	0.7	−0.014 (−0.075; 0.046)	0.742	L-shaped
Pop16	3	7.38	0.69	0.68	−0.019 (−0.073; 0.033)	0.996	L-shaped
Pop17	5	8.26	0.68	0.74	0.075 (0.022; 0.126)	0.868	L-shaped
Mean	4.5 ( ± 3.2)	8.13 ( ± 0.66)	0.68 ( ± 0.04)	0.71 ( ± 0.04)	0.04 (0.03)	–	–

Mean: mean estimation of the genetic diversity parameters and standard deviation (between parenthesis).

Ap number of private alleles, Ar allelic richness, H_O_ e H_E_ observed and expected heterozygosity, f fixation index. TPM unicaudal probability of excess heterozygotes between microsatellites following the two-phase mutation model.

We did not detect signal of bottleneck in any population, since the unicaudal probability for the excess of heterozygotes between microsatellite markers was not significant (P > 0.7) and the proportion of alleles in the frequency intervals followed an L distribution format (**Table 1**).

### Environmental Landscape and Local Variable

**Figure 2:**
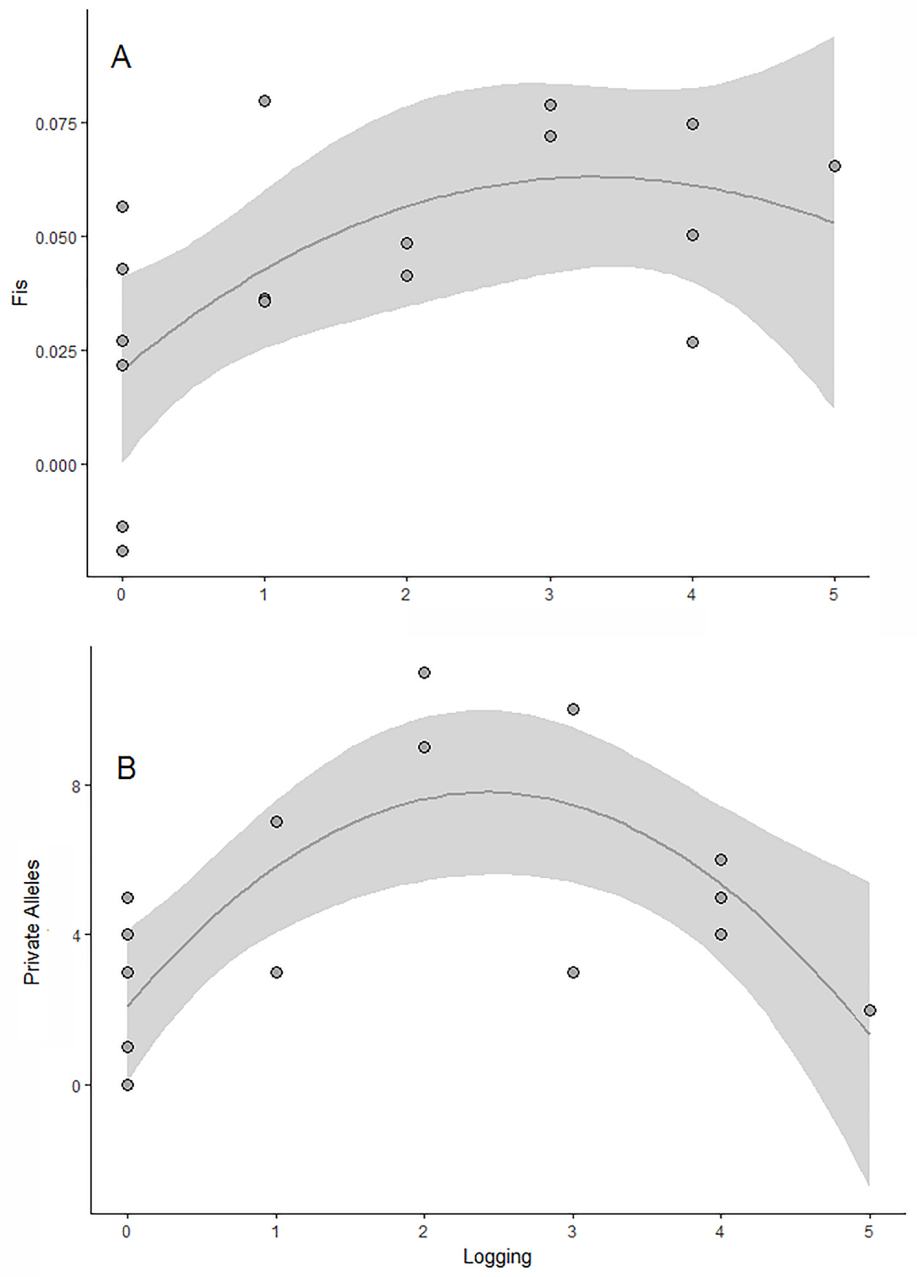
Effects of logging (number of stumps) on the genetic diversity of *E. edulis* populations. **(A)** private alleles; **(B)** fixation index (*f*). The gray area corresponds to the confidence interval of the generalized additive models.

The scale of effect that strongly influences the genetic parameters varied in relation to the response variable and also to the landscape metric (**Table S1**). The observed and expected allelic richness and heterozygosity were not affected by any landscape or local variable (null model selected; **Table 2**). In contrast, the number of private alleles and the coefficient of inbreeding were significantly affected by logging intensity within the fragment (**Figure 2**). The concurvity measures were very small in the selected models, suggesting negligible concurvity (estimate < 0.0005).

**Table 2:** Result of the multimodel inference using generalized additive models (GAM) for the different parameters of genetic diversity. In all cases, we include the null model, containing only the intercept and error parameters.

	Model	ΔAICc	Df	*Wi*
Ar	**Null**	**0**	**2**	**0.418**
	P.I.	1.6	3.37	0.186
	Log	1.9	3	0.159
	F.C.	2.7	3	0.109
	P.I. + log	3.8	4.01	0.061
	F.C. + log	4.9	4	0.036
	F.C. + P.I.	5.2	4.38	0.031
	Full	7.9	5.01	0.008
Ap	**Log**	**0**	**4.16**	**0.686**
	F.C. + log	3.8	5.09	0.103
	P.I. + log	4.1	5.11	0.09
	Null	5.1	2	0.054
	P.I.	6.1	3	0.032
	F.C.	6.7	3	0.024
	F.C. + P.I.	8.4	4	0.01
	Full	20.7	9.7	0.001
H_O_	F.C.	0	3.95	0.296
	**Null**	**0.2**	**2**	**0.262**
	P.I.	1.1	3	0.173
	Log	2.4	3	0.091
	F.C. + P.I.	2.5	4.90	0.085
	P.I. + log	3	4	0.066
	F.C. + log	4.8	5.95	0.027
	Full	9.2	7.45	0.003
H_E_	**Null**	**0**	**2**	**0.342**
	P.I.	0.3	4.74	0.293
	F.C. + P.I.	2.1	4	0.122
	F.C.	2.6	3	0.095
	Log	2.9	3	0.081
	P.I. + log	3.5	5.78	0.058
	Full	6.5	6.74	0.013
	F.C. + log	7.1	5.73	0.010
*f*	**Log**	**0**	**3.42**	**0.582**
	Null	2.8	2	0.145
	F.C. + log	3.5	4.32	0.103
	P.I. + log	3.6	4.36	0.097
	P.I.	5.7	3	0.034
	F.C.	5.8	3	0.033
	Full	7.7	5.27	0.012
	F.C. + P.I.	9.2	4	0.006

ΔAICc value of the Akaike Information Criterion corrected for small sample size, df number of parameters of the models and Wi Akaike’s weight. Ar, allelic richness; Ap, number of private alleles; H_O_ and H_E,_ observed and expected heterozygosity; f, fixation index; F.C., forest cover; P.I., proximity index; Log, logging intensity. Bold texts correspond to the best models selected for each genetic parameter.

### Genetic Structure

**Figure 3:**
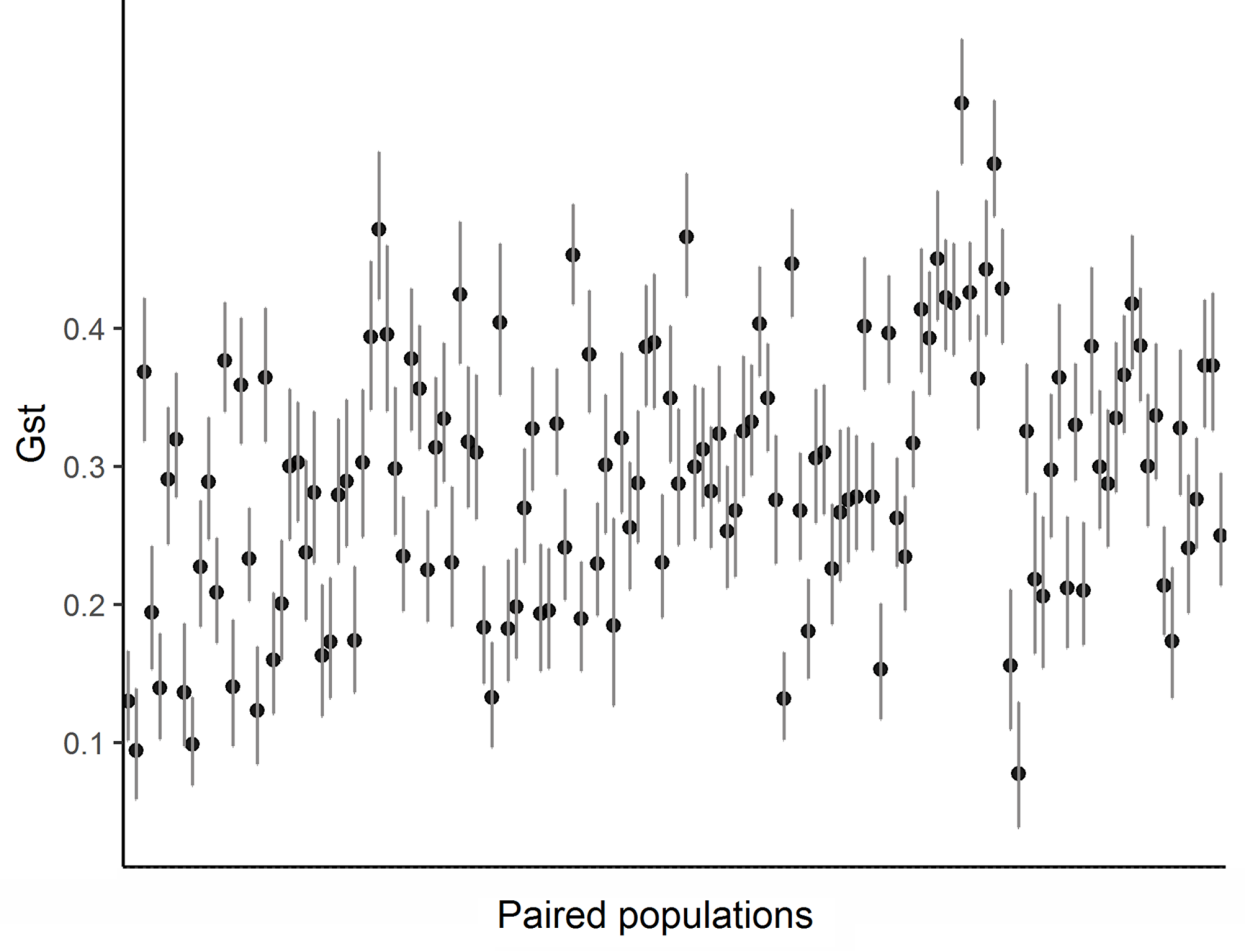
The G_ST_ values paired among the 17 populations of *E. edulis* located in the Atlantic Forest of Southern Bahia.

**Figure 4:**
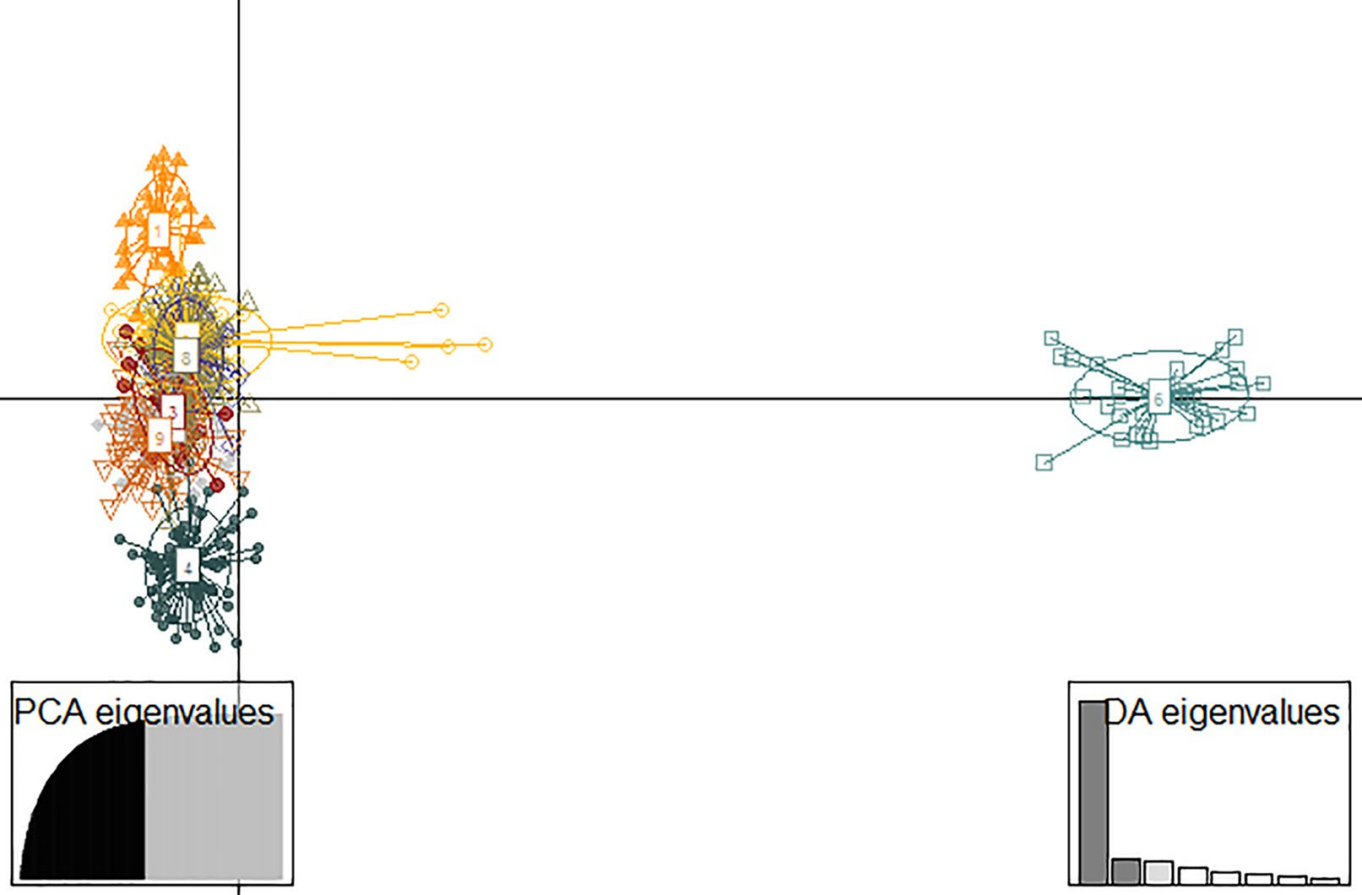
Discriminant analysis of principal components (DAPC) for 17 populations of *E. edulis* from the Atlantic Forest of Southern Bahia, Brazil. The dots symbolize individuals within the population and the circles represent the genetic clusters defined by the DAPC.

Paired G_ST_ ranged from 0.077 to 0.563 (0.292 ± 0.095). All estimates of genetic differentiation given by this parameter were significantly different from zero (**Figure 3**). Estimated values for paired F_ST_ ranged from 0.022 to 0.19 (0.089 ± 0.032) and although genetic differentiation between populations was less pronounced than that presented by G_ST_ (**Table S4**), we also found a significant genetic structure pattern through this analyze. The ideal number of genetic groupings indicated by the lowest BIC in the DAPC analysis was 9 (K9 = 1,048.768) (**Figure S1B**). However, only three clusters were formed by a clear genetic differentiation (populations 6, 15 e 16) (**Figure 4**). The other groups were composed of different proportion of individuals that belonged to distinct geographic populations, which indicates a great mixture of the gene pool of those populations.

## Discussion

Multilevel approach studies have been increasing in landscape ecology literature in recent years (Graham and Blake, 2001; Buskirk, 2005; Calamari et al., 2018), but are still scarce in the genetic field (González-Fernández et al., 2019). Our study represent a step forward in filling this gap, since it simultaneously evaluated the impacts of human activities at landscape and local scales on the genetic diversity of *E. edulis* populations in the Atlantic Forest. Despite the demographic decline recorded in many localities of *E. edulis* occurrence (Galetti and Fernandez, 1998; Matos and Bovi, 2002), we did not find evidence of a genetic bottleneck in any of the populations sampled. However, we found that the number of effective alleles and the fixation index were influenced by logging activity. In addition, we found evidence of genetic structure among the populations investigated suggests a limitation on gene flow that operates at larger scales.

The geographic distribution of *E. edulis* was originally recorded in almost all Atlantic Forest biome, with high densities in subcanopy of dense ombrophilous forests (Reis et al., 2000a). The current threat status of *E. edulis* is vulnerable to extinction, mainly due to the 30% population decline registered in the last six decades (Martinelli and Moraes, 2013). In spite of that, our study revealed that the populations investigated showed no signs of recent bottlenecks and still retain high genetic diversity. This result is compatible with previous studies (Conte et al., 2008; Carvalho et al., 2015; Santos et al., 2015), and also corroborates the hypothesis that recent population declines are not sufficient to produce changes in genetic diversity levels (Klank et al., 2012; Montes et al., 2016). This is because although genetic bottleneck events result commonly in the loss of rare alleles—frequency < 0.05—(Allendorf, 1986), heterozygosity levels are poorly influenced since these alleles contribute little to the formation of heterozygous individuals (Hartl and Clark, 1997). Therefore, our results suggest that *E. edulis* populations reflect a historical condition of the original species distribution since it corresponds to the expected diversity pattern for populations with a broad geographic distribution (Hamrick et al., 1992).

Contrary to our initial expectations, we found that only local scale disturbances affect some estimates of genetic diversity. Previous studies suggested the importance of landscape scale effects on the genetic diversity. For instance, a simulation study by Jackson and Fahrig (2014) found that while species abundance is influenced by local scale disturbances, genetic diversity tends to be more affected by landscape composition and configuration at large spatial scales. In addition, Carvalho et al. (2015) assessing the effects of recent landscape changes on genetic diversity of the same species, *E. edulis*, found the resistance of the matrix as one of the main factors affecting the allelic richness of these populations. These authors conducted the study in the southeastern Atlantic Forest, thus a possible explanation for the lack of landscape effects on our study, may be attributed to differences in land-use history between regions. The fragmentation of the southeastern Atlantic Forest began with coffee plantations in the 19th century (Dean, 1996). In contrast, intensive deforestation in our region began only during the 1980s (Rocha, 2006). A meta-analysis by Schlaepfer et al. (2018) identified that the age of anthropogenic fragmentation is a determining factor to detect genetic diversity loss. Thus, we believe that the recent landscape modification in the southern Bahia still did not trigger a genetic diversity erosion in *E. edulis* populations.

On the other hand, we observed a faster negative effect of logging activity, on the number of private alleles and fixation index. The inverted U-shaped relationship between logging and the number of private alleles showed that both areas with low and high logging activity had few alleles, which are unshared among populations. One possible explanation for this pattern is that the small number of private alleles in the poorly explored areas reflected the historical condition of the geographic distribution of *E. edulis*, with great populations and high level of gene flow (Gaiotto et al., 2003, Santos et al., 2015). In contrast, the low number of private alleles in intensively exploited areas was probably the result of genetic drift, which is a major cause of random loss of alleles in reduced populations (Frankham et al., 2002).

The relationship between logging and fixation index was expressed by a non-linear relationship, evidencing an increasing inbreeding with logging intensification. The results obtained are in agreement with the expected pattern for the traditional harvest system of this species that compromises the demographic structure of the populations by leaving only a few reproductive individuals in the fragments. (Reis et al., 2000a). In these scenarios an inbred pattern is common because the natural regeneration of populations is associated with the presence of few matrices (Murawski et al., 1994). On the other hand, in efficient forest restoration practices in which several matrices of distinct geograpthical origins is used the inbred patterns can be diluted (Zucchi et al., 2018). The genetic structure of *E. edulis* populations showed a significant difference in the gene pool of all populations compared. In addition, we found that 3 out of 17 populations investigated had completely distinct gene pools due to the low probability of sharing common alleles among them. This result is in agreement with Santos et al. (2015; 2016) who investigated the effect of habitat loss on the genetic structure of *E. edulis*, and recorded that deforestation caused a decline in the distance of gene flow of juvenile individuals. In addition, a recent study with the *Oenocarpus bataua* palm tree found that habitat loss and fragmentation were mainly responsible for increasing structure and reducing genetic diversity in female gametes of this species (Browne and Karubian, 2018). Thus, the limitation of pollen exchange and especially in processes involving long-distance seed dispersal should be the main reason for the strong genetic structure found in our populations, since currently there are only 12% of the original habitat for this species (Ribeiro et al., 2009). Finally, we found no evidence of spatial genetic structure on a fine scale distance. This result was already expected, because *E. edulis* is a predominantly allogenic, pollinated and dispersed by a large number of organisms that can move at long distances (Gaiotto et al., 2003; Dorneles et al., 2013; Galetti et al., 2013).

## Conclusions and Conservation Implications

To conclude, our study revealed that the assessment of local and landscape-scale anthropogenic pressures may provide different information about the genetic vulnerability of a species. Our findings showed that intensification of logging activities affected *E. edulis* genetic diversity more rapidly than recent landscape modifications. However, the strong genetic structure found in all populations in our study suggests a limitation of gene flow resulting from habitat loss on a regional scale. Although current populations of *E. edulis* still maintain high levels of genetic diversity, this situation tends not to be maintained if local and landscape-scale anthropogenic pressures are not attenuated. This is because the loss of private alleles and increased inbreeding, as found in our results, can affect the suitability and adaptability of *E. edulis* populations if kept small and isolated for many generations. For this reason, to maximize conservation efforts for species of long-term economic interest, we recommend increasing inspection to reduce illegal exploitation of these species. In addition, we also suggest investments in reforestation of degraded areas in order to increase gene flow among Atlantic Forest fragments and rebalance inbreeding levels in other populations.

## Data Availability Statement

The SSR genotypes are available on the Dryad Digital Repository under https://doi.org/10.5061/dryad.ghx3ffbjh.

## Author Contributions

All authors contributed to the conception of the work. Conceived the study design: LAS, EC, FG. Data collection for the manuscript: LAS, DF, LRS. Wrote the manuscript: LAS. Provided input and revised the manuscript: LAS, EC, LRS, DF, FG. All authors approved the final manuscript.

## Funding

The authors thank Conselho Nacional de Desenvolvimento Científico e Tecnológico (CNPq) (Grant N° 426828/2016-0) and Universidade Estadual de Santa Cruz (UESC) (Grant N° 00220.1100.1681) for financial support. We also thank for grants to LAS (Fapesb—Ph.D.), EC and FG (CNPq-PQ), and LRS (Capes-PNPD).

## Conflict of Interest

The authors declare that the research was conducted in the absence of any commercial or financial relationships that could be construed as a potential conflict of interest.
