# Supplementary material for: Anthropogenic Disturbances Eroding the Genetic Diversity of a Threatened Palm Tree: A Multiscale Approach
Source: Front Genet. 2019 Nov 7;10:1090. doi: 10.3389/fgene.2019.01090 (PMC6855268; doi:10.3389/fgene.2019.01090)
Supplement: Supplementary file 1 [file DataSheet_1.pdf]

Supplementary Material

Supplementary Figure Legends:

Supplementary Figure 1. Initial stages of the Discriminant analysis of principal components (DAPC). **(A)** cumulative variance explained by the principal component analysis relative to the number of components (PCs) retained; **(B)** selection of the optimal number of clusters in the DAPC using the lowest value of the Bayesian information criterion (BIC); **(C)** cross-validation procedure to choose the optimal number of principal components for DAPC.

Suplementary Table:

Table S1. Characterization of the composition (forest cover) and configuration (proximity index) of the landscape in the six spatial scales analyzed in this study (0.5 - 2.0 km) and quantification of logging intensity for the 17 Atlantic Forest fragments sampled in southern Bahia.

Fragments	**Landscape-scale: measures quantified in 6 buffers with different radius size (km)**														**Local scale**
	Mean Proximity index							Forest cover (%)							Logging Intensity
	0.5	0.75	1	1.25	1.5	1.75	2	0.5	0.75	1	1.25	1.5	1.75	2	
Frag 1	0	0	0	0	0	0	734.7	100	100	100	100	100	99.5	95.8	0
Frag 2	0	0	820.5	0	2266.5	434.9	3312.5	97.8	97.8	97.0	96.0	92.4	88.6	85.4	4
Frag 3	0	0	3340.3	0	0	551.3	4588.2	89.8	84.0	78.5	78.7	80.0	80.9	79.5	1
Frag 4	0	16.3	80.1	168.9	286.0	630.9	1606.7	77.4	65.6	66.5	71.3	73.7	73.4	71.0	0
Frag 5	0	4.5	17.4	119.3	516.4	362.7	456.2	73.9	61.0	59.9	61.0	60.5	62.6	63.7	1
Frag 6	0	0	0	393.1	351.4	261.8	253.1	97.1	84.6	76.7	72.1	68.4	66.1	64.0	2
Frag 7	27.9	4.3	33.2	721.5	242.1	487.2	1723.6	45.5	47.9	49.2	52.2	53.4	56.3	61.2	2
Frag 8	0	0	95.6	361.5	1725.5	246.9	358.3	85.1	72.6	60.4	58.0	55.7	52.5	51.9	4
Frag 9	0	0	1215.5	1986.2	1523.3	1333.1	1016.8	93.9	90.3	80.4	69.1	59.5	53.7	51.4	3
Frag 10	0	127.4	187.8	68.8	157.4	232.4	471.0	61.4	50.1	40.8	37.9	43.2	46.6	49.8	0
Frag 11	784.1	1663.8	1158.1	1725.5	1460.1	1169.9	1293.1	79.9	62.1	53.3	51.2	48.3	44.8	45.6	3
Frag 12	7.3	12.1	19.7	796.8	19.5	37.0	92.2	61.3	55.8	51.0	48.1	46.1	42.3	41.8	0
Frag 13	0	266.8	830.5	1195.5	1030.9	1056.7	1033.6	65.4	64.6	58.7	54.7	48.3	45.0	43.0	1
Frag 14	1.4	99.8	15.7	219.8	93.1	75.0	110.0	3.5	52.1	21.6	44.4	31.3	43.1	42.5	5
Frag 15	0	310.6	433.2	554.2	455.3	342.8	175.7	78.4	63.3	52.8	46.1	40.4	38.4	37.0	0
Frag 16	10.6	13.4	46.0	49.5	18.8	53.1	57.0	39.4	22.1	20.1	25.9	28.5	26.9	27.1	0
Frag 17	0	42.8	10.8	0	57.2	0.5	2.6	28.7	18.2	17.7	21.5	22.7	21.7	18.6	4

Table S2. Pearson correlation values for descriptive variables of landscape composition and configuration quantified on seven spatial scales. F.C. forest cover, E.D. edge density, P.S. mean patch size and P.I. proximity index,

Scale (km)	Pearson correlation (r)					
	F.C./ E.D	F.C./P.S.	F.C./M.P.I.	E.D/P.S.	E.D/M.P.I.	P.S./M.P.I
0.5	-0.32	0.92	0.087	-0.45	0.47	-0.2
0.75	-0.34	0.87	-0.063	-0.64	0.66	-0.31
1	-0.26	0.73	0.35	-0.63	0.32	-0.004
1.25	-0.33	0.8	-0.053	-0.61	0.72	-0.42
1500	-0.41	0.82	0.26	-0.59	0.35	-0.1
1.75	-0.43	0.8	0.059	-0.63	0.72	-0.24
2	-0.33	0.75	0.62	-0.62	0.11	0.22

Table S3. Scale selection reflecting the strongest interaction between genetic parameters and landscape metrics (effect scale) with significant values of the spatial autocorrelation test by Moran's index. **Ar** allelic richness, **Ap** number of private alleles, $\mathbf{H_O}$ e $\mathbf{H_E}$ observed and expected heterozygosity, ***f*** fixation index.

Model	Genetic parameter	Landscape metrics	Scale of effect (km)	Moran
GAM	Ar	forest cover	1.75	0.314
GAM	Ap	forest cover	0.75	0.626
GAM	H_O	forest cover	0.5	0.457
GAM	H_E	forest cover	1.5	0.861
GAM	*f*	forest cover	2	0.816
GAM	Ar	proximity index	1	0.643
GAM	Ap	proximity index	1.25	0.934
GAM	H_O	proximity index	1.25	0.316
GAM	H_E	proximity index	1.5	0.569
GAM	*f*	proximity index	1	0.777

Table S4. Genetic differentiation estimated by G_{ST} (dark grey) and F_{ST} (light gray) values for all pairs of the 17 *E. edulis* populations sampled in southern Bahia.

**Populations**	Pop 1	Pop 2	Pop 3	Pop 4	Pop 5	Pop 6	Pop 7	Pop 8	Pop 9	Pop 10	Pop 11	Pop 12	Pop 13	Pop 14	Pop 15	Pop 16	Pop 17
Pop 1		0.033	0.074	0.097	0.077	0.135	0.047	0.107	0.070	0.042	0.029	0.108	0.058	0.052	0.098	0.112	0.043
Pop 2	0.099		0.060	0.103	0.083	0.152	0.059	0.105	0.064	0.055	0.037	0.110	0.050	0.069	0.085	0.107	0.056
Pop 3	0.227	0.196		0.107	0.098	0.154	0.091	0.086	0.077	0.088	0.052	0.087	0.069	0.099	0.118	0.125	0.066
Pop 4	0.289	0.331	0.349		0.047	0.152	0.085	0.052	0.087	0.097	0.073	0.075	0.091	0.117	0.127	0.116	0.081
Pop 5	0.208	0.241	0.287	0.132		0.149	0.091	0.074	0.100	0.103	0.072	0.083	0.086	0.107	0.139	0.102	0.050
Pop 6	0.377	0.453	0.466	0.447	0.397		0.149	0.158	0.132	0.135	0.123	0.137	0.128	0.157	0.190	0.152	0.117
Pop 7	0.140	0.190	0.299	0.268	0.262	0.443		0.092	0.059	0.047	0.022	0.090	0.061	0.073	0.094	0.119	0.062
Pop 8	0.359	0.381	0.312	0.181	0.234	0.519	0.330		0.084	0.103	0.076	0.071	0.082	0.115	0.117	0.115	0.078
Pop 9	0.233	0.229	0.282	0.306	0.317	0.429	0.210	0.337		0.058	0.044	0.081	0.059	0.088	0.106	0.111	0.065
Pop 10	0.130	0.183	0.301	0.324	0.310	0.414	0.156	0.387	0.214		0.033	0.096	0.042	0.068	0.091	0.096	0.066
Pop 11	0.094	0.132	0.185	0.253	0.226	0.393	0.077	0.300	0.173	0.123		0.070	0.041	0.056	0.080	0.088	0.046
Pop 12	0.368	0.404	0.320	0.268	0.267	0.450	0.326	0.287	0.328	0.364	0.281		0.073	0.122	0.130	0.116	0.077
Pop 13	0.194	0.182	0.256	0.325	0.275	0.423	0.218	0.335	0.241	0.160	0.163	0.303		0.074	0.105	0.104	0.058
Pop 14	0.139	0.198	0.288	0.332	0.278	0.418	0.206	0.366	0.276	0.201	0.173	0.394	0.235		0.110	0.123	0.076
Pop 15	0.290	0.270	0.386	0.403	0.402	0.563	0.297	0.418	0.373	0.300	0.279	0.472	0.378	0.313		0.140	0.094
Pop 16	0.320	0.327	0.390	0.350	0.278	0.426	0.364	0.387	0.373	0.303	0.289	0.396	0.356	0.335	0.425		0.096
Pop 17	0.136	0.193	0.230	0.275	0.153	0.364	0.212	0.300	0.250	0.238	0.174	0.298	0.225	0.230	0.318	0.310	
