# Supplementary figures and images for: Anthropogenic Disturbances Eroding the Genetic Diversity of a Threatened Palm Tree: A Multiscale Approach
Source: Front Genet. 2019 Nov 7;10:1090. doi: 10.3389/fgene.2019.01090 (PMC6855268; doi:10.3389/fgene.2019.01090)

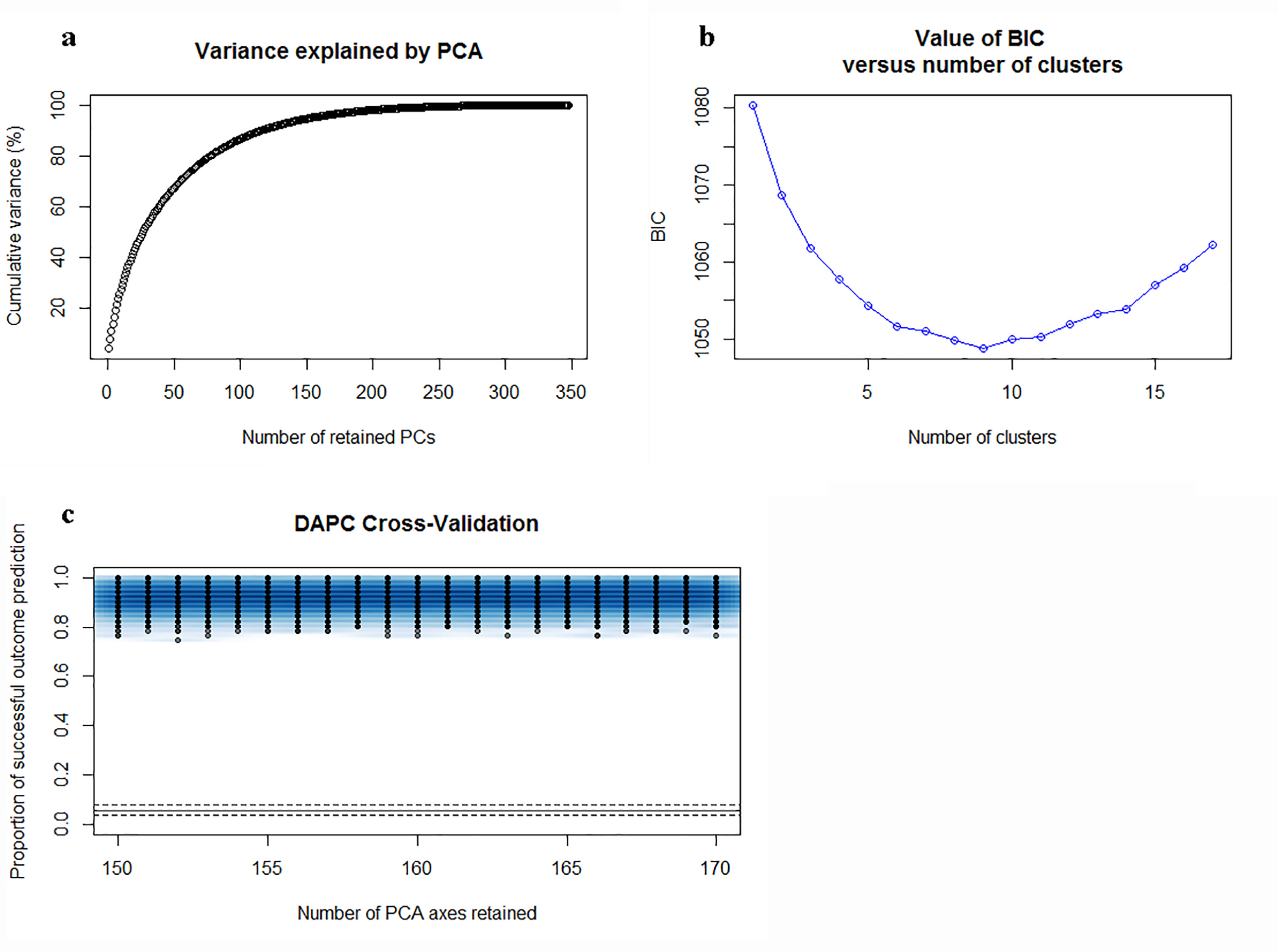

Supplement: Supplementary file 2 [file Image_1.tif]
